# Number of Published Randomized Controlled Multi Center Trials Testing Pharmacological Interventions or Devices Is Increasing in Both Medical and Surgical Specialties

**DOI:** 10.1371/journal.pone.0101383

**Published:** 2014-07-14

**Authors:** Anne Kjaergaard Danielsen, Cecilie Okholm, Hans-Christian Pommergaard, Jakob Burcharth, Jacob Rosenberg

**Affiliations:** 1 Department of Nursing, Faculty of Health and Technology, Metropolitan University College, Copenhagen, Denmark; 2 Department of Surgery, Herlev Hospital, University of Copenhagen, Herlev, Denmark; Cardiff University, United Kingdom

## Abstract

**Background:**

In general, there is a need for testing new interventions in large randomized controlled trials. Depending on the research question it may be advantageous to establish multicenter studies as a way of organizing clinical trials in order to increase study power.

**Main Objectives:**

The object of this study was to investigate the development in the organization of multicenter studies, the distribution of studies within different clinical specialties, across continents, and investigate the differences related to testing various interventions.

**Methods and Materials:**

A literature search was done in MEDLINE for multicenter studies published in 1995, 2000, 2005, and 2010, respectively. Data extraction identified data related to clinical specialties, interventions, participating patients, departments, countries, and continents.

**Results:**

The number of multicenter studies increased from 112 in 1995 to 1,273 in 2010, with a larger share of multicenter studies being performed in Europe and North America. The pharmacological interventions were primarily being tested in medical studies followed by the device tests predominantly in surgical studies. The number of included patients as well as the number of participating departments increased during the time span, though the increase in studies was most evident in Europe and North America compared with the rest of the world.

## Introduction

In general, results relying on meta-analyses of randomized controlled trials are being regarded as the highest level of evidence [1]. Hence, the future of clinical research and therefore the future of medical decision-making should be based on such studies if possible [2]. Moreover, for a clinical research protocol to be ethically sound the medical methods must be valid and clinically feasible, and the study should be designed to obtain sufficient power [3].

When new treatments are introduced without having been tested in high quality randomized controlled trials there seems to be methodological problems in some clinical research areas [4], [5]. This may rely on specific problems related to achieving sufficient power, and as we at the moment are faced with several methodological and practical problems in the surgical research community [6], we assumed that these problems might be present in other clinical specialties as well. The before mentioned problems cover lack of research on the surgical procedures itself, and maybe a decline in the number of randomized controlled trials (RCTs) being performed within clinical therapy [7], although, a recent study pointed at an increase in the publication of surgical RCTs [8]. These problems could be relevant for studies testing pharmacological interventions, medical devices, or surgical procedures, as well [9].One of the key problems may be related to inclusion of sufficient participants in the clinical trials. An increase in sample size would increase the probability of producing a precise and dependable evaluation of the efficacy of the intervention under study.

The above mentioned hypotheses have not been explored in a systematic way uncovering aspects related to obtaining sufficient power in clinical research in multicenter studies. Our hypothesis before initiating the study was that we expected studies originating from the medical specialty would be larger in number than studies reporting results from surgery, psychiatry and general practice. Furthermore, we expected studies testing pharmacological interventions would also be larger in number. The object of this study was therefore to investigate the development in clinical studies organized as multicenter studies from 1995 to 2010. Moreover, we wanted to explore this issue related to the distribution of studies within different clinical specialties, and across continents. Furthermore, we wanted to investigate the differences in numbers of the published papers, and in participants included in the studies, and in relation to the various interventions being tested.

## Materials and Methods

**Figure 1:**
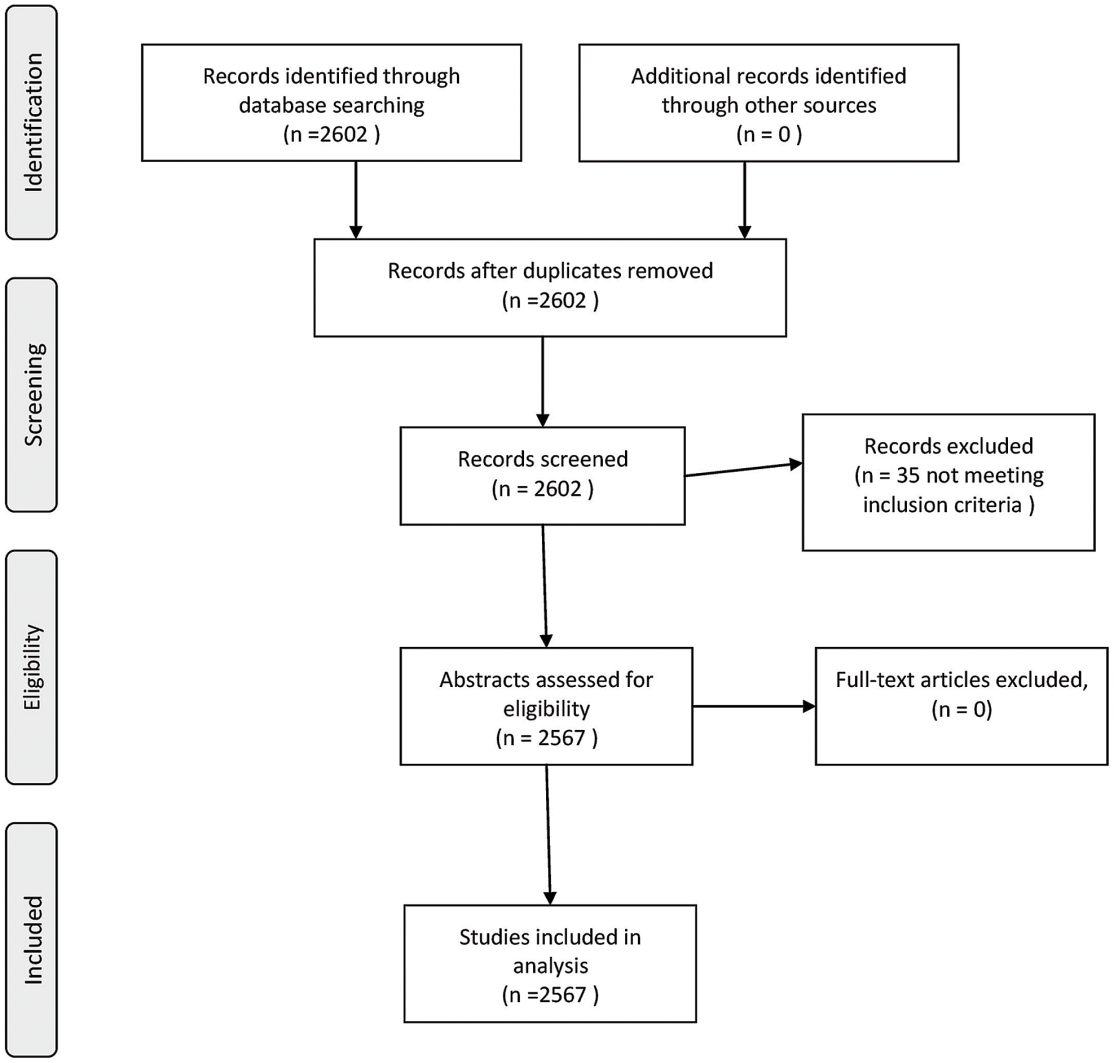
Flow chart.

**Figure 2:**
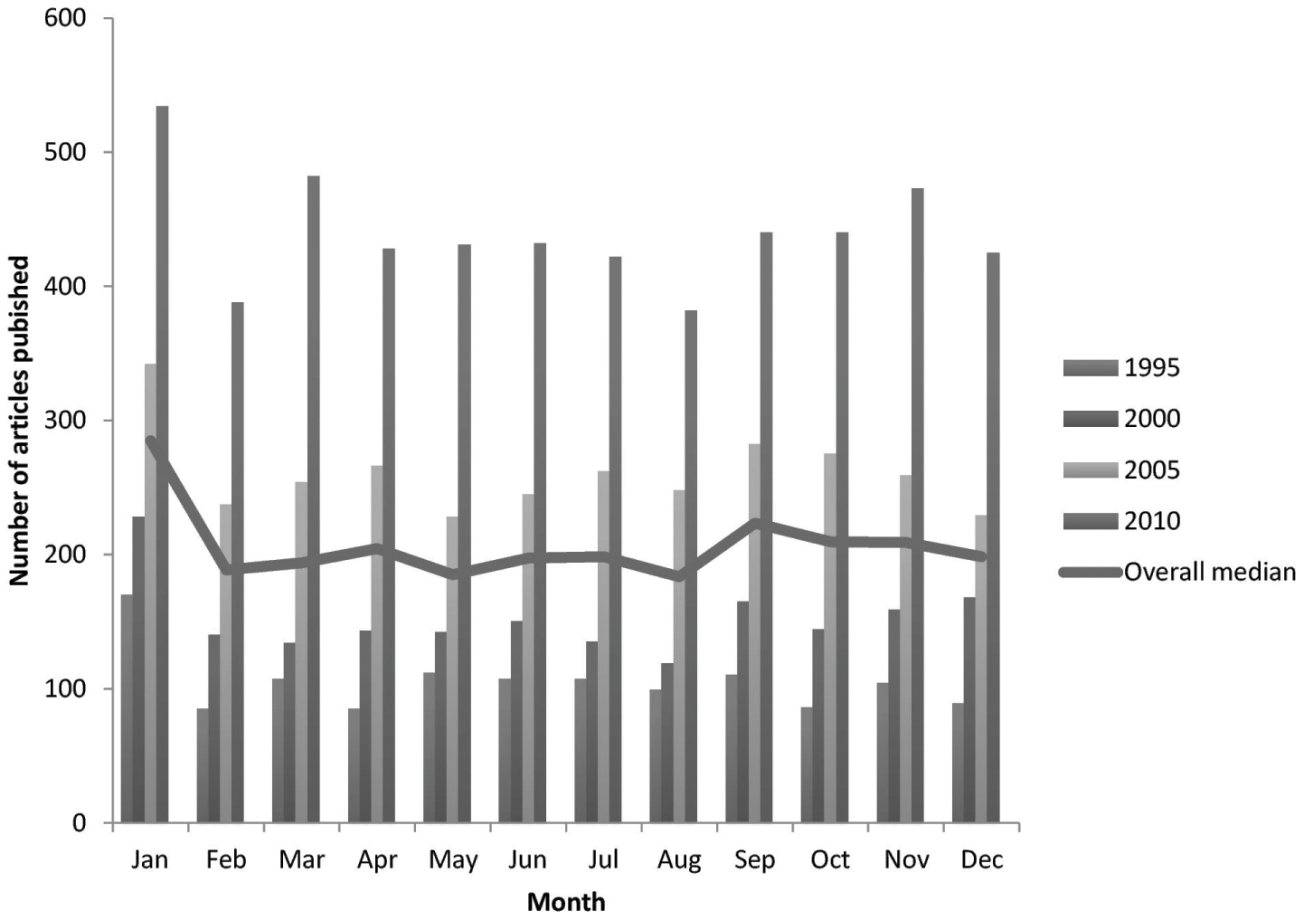
Showing publication rates for 1995, 2000, 2005 and 2010, and the overall median of the rates.

We searched for published studies (Figure 1) in MEDLINE with the following search terms: “Multicenter Study”(Publication Type) OR ((((((multicenter study(Title/Abstract) OR multicentre study(Title/Abstract)) OR multicenter studies(Title/Abstract)) OR multicentre studies(Title/Abstract)) OR multi-center study(Title/Abstract)) OR multi-centre study(Title/Abstract)) OR multi-center studies(Title/Abstract)) OR multi-centre studies(Title/Abstract). We used the limiters: abstracts available, humans, RCT, English, and furthermore we narrowed the search by only including papers that were published in the months February, June, and November within five-year intervals from 1995 to 2010. An overview of the publication rates in these months revealed that they were considered representative of the publication rates for a whole year (Figure 2).

Inclusion criteria were: papers reporting RCTs that were organized as multicenter studies, and testing clinical interventions aimed at humans. Exclusion criteria were non-randomized designs, odontologic studies, protocol abstracts, and studies with less than two participating centers.

Data were extracted by one of the authors (CO) from abstracts including the following variables: clinical specialty (medicine, surgery, psychiatry, and general practice), medical subspecialties (cardiology, gastroenterology, endocrinology, oncology, pulmonary medicine, and “others” covering e.g. neurology, pediatrics, hematology) as well as surgical subspecialties (thoracic surgery, gastrointestinal (general) surgery, vascular surgery, urology, orthopedic surgery, and “others” covering e.g. ophthalmology, gynecology, neurosurgery). Furthermore, we registered continent of origin, number of participating countries, number of participating centers, the tested intervention (device, drug, observation, and others), and number of included patients if available. If reading the abstracts could not retrieve data on the number of patients, full-text papers were obtained.

Data were analyzed using SPSS 19 (IBM Corp. Released 2010. IBM SPSS Statistics for Windows, Version 19.0. Armonk, NY: IBM Corp). We did not do any statistical tests as our data, although large in number, would be better presented descriptively. Data describing number of participants and studies were analyzed as continuous variables, and data describing specialties and subspecialties were handled as categorical variables. Hence, results were presented using mean and standard deviation and median and min-max, and percentages where relevant.

The study was exempt from approval with the Danish Ethical Committee as well as the Danish Data Protection Agency, as we did not include any form of biomedical intervention or any personal data related to individual and identifiable humans.

## Results

The search in the database for relevant studies published in the months of February, June, and November in 1995, 2000, 2005, and 2010, identified 2,602 studies. Based on reading of the abstracts we included 2,567 studies and excluded 35 studies not meeting our inclusion criteria (Figure 1). An examination of missing data in the included studies revealed that some of the selected variables were sparsely reported (number of participating countries: missing data 5.7%, number, or participating departments: missing data 81% and number of included participants: missing data 2.9%). However, the major part of the variables was reported in 100% of the cases.

### Number and percentage of trials organized in multicentre studies

**Figure 3:**
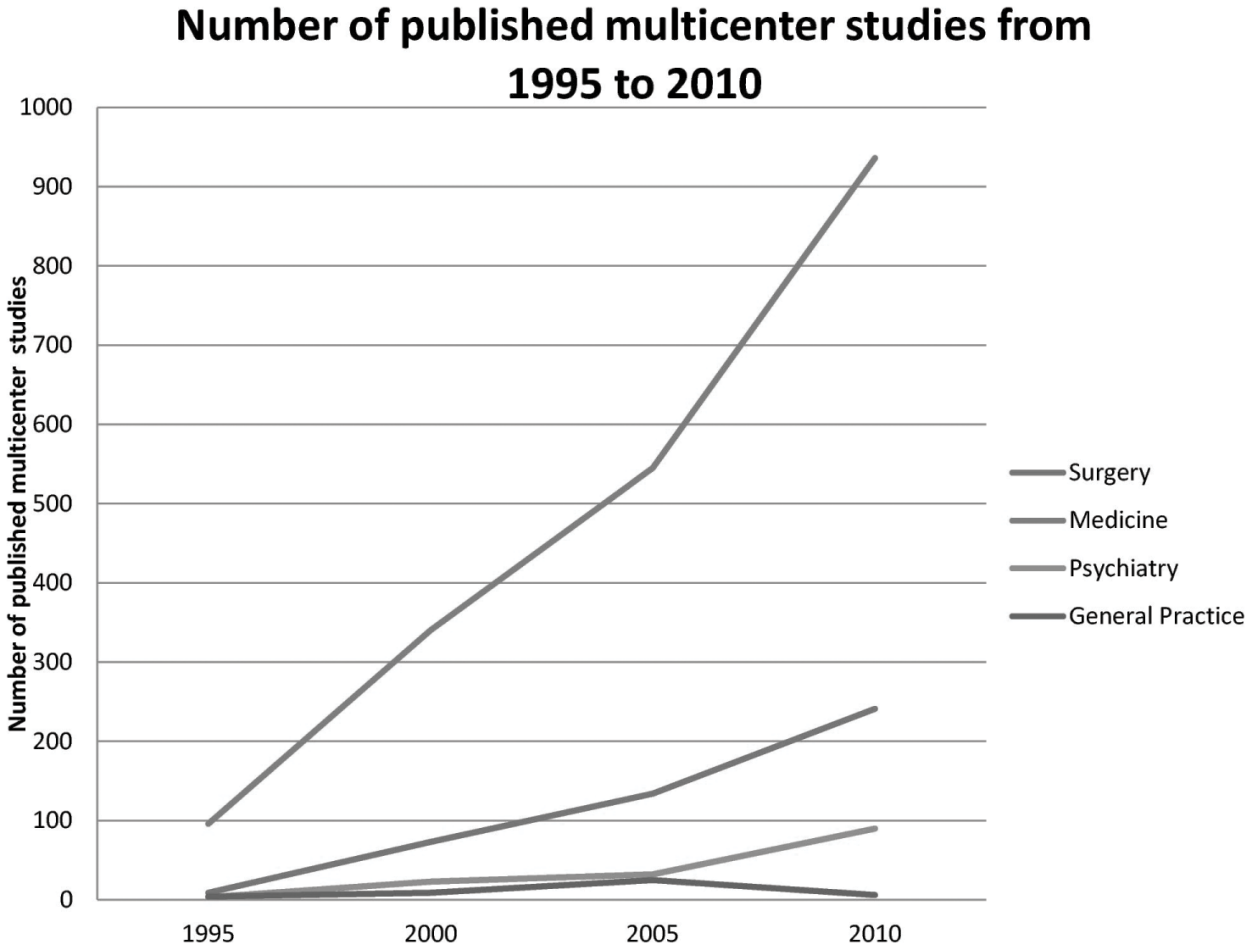
Number of published multicenter studies from 1995 to 2010.

**Figure 4:**
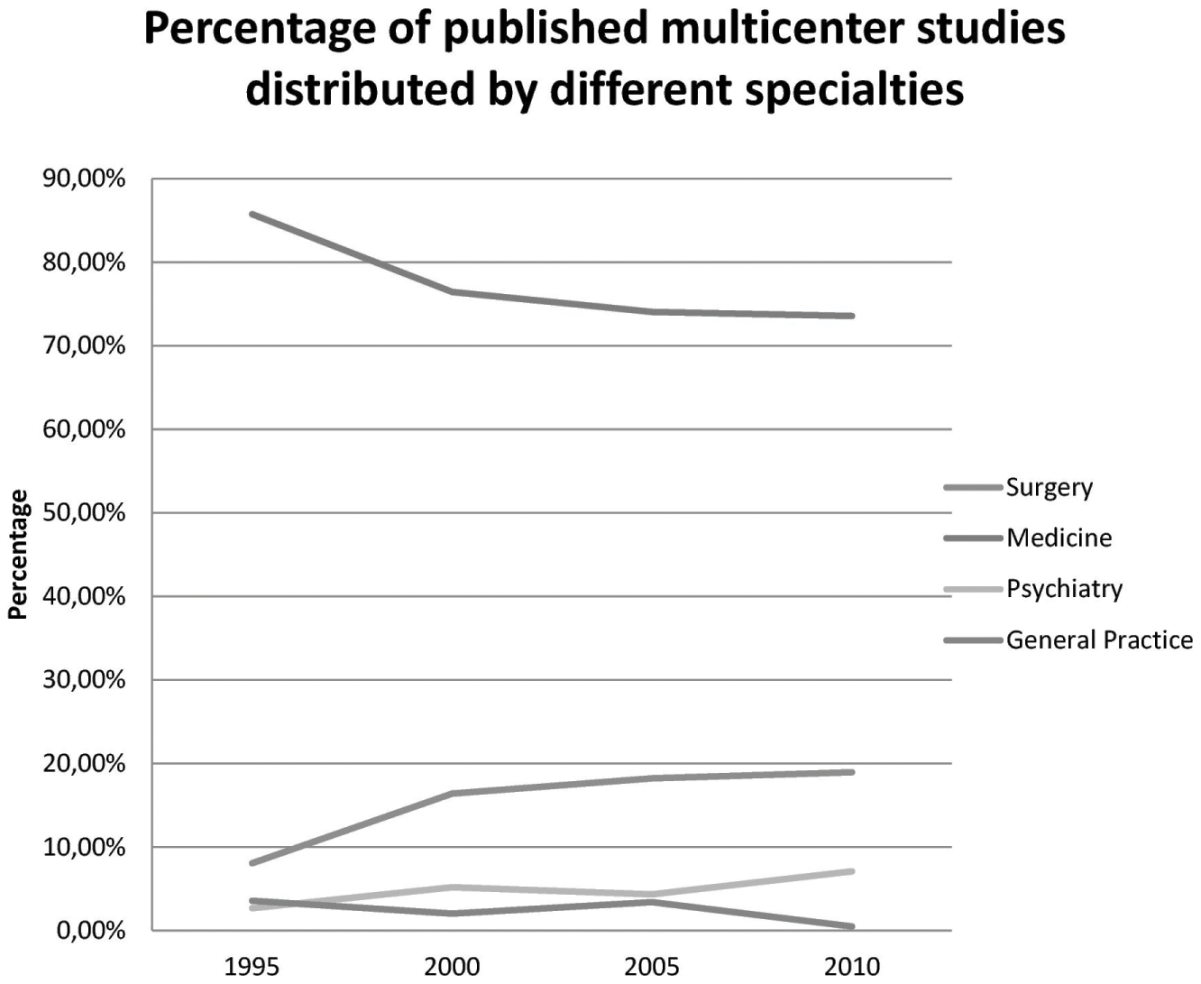
Percentage of published multicenter studies distributed by different specialties.

The results showed a substantial increase in the total number of trials in all specialties organized and published as multicenter studies with 112 reported trials in 1995 and 1,273 trials reported in 2010. When differentiating between the specialties it was evident that more studies in numbers originated from medical specialties compared with other specialties (Figure 3). However, the relative distribution in percent of the individual specialties only varied very little over time (Figure 4).

### Number of studies distributed by medical or surgical subspecialty

When exploring the publication trend within the different medical and surgical subspecialties, we found a rise in multicenter publications from 1995 to 2010 within all medical subspecialties (cardiology; n = 25 to n = 151, oncology; n = 25 to n = 141, endocrinology; n = 5 to n = 106, gastroenterology; n = 10 to n = 60, pulmonary medicine; n = 5 to n = 48). When looking at the surgical subspecialties the development in numbers was equally positive, and all the surgical subspecialties experienced an increasing number of multicenter publications from 1995 to 2010 (gastroenterological surgery; n = 4 to n = 28, thoracic surgery; n = 0 to 12, orthopedic surgery; n = 0 to n = 20, vascular surgery; n = 0 to n = 27, urology; n = 3 to n = 100).

### Number of studies distributed by continent

The number of studies being organized as multicenter studies across continents pointed at Europe and North America accounting for the largest increase (Europe 48 (year 1995) – 555 (year 2010), North America 52 (year 1995) – 491 (year 2010)). Furthermore, Europe and North America combined covered more than 80% of the multicenter studies published in 2010, and including 89% of the total number of participants.

### Number of participants included in multicentre studies

**Figure 5:**
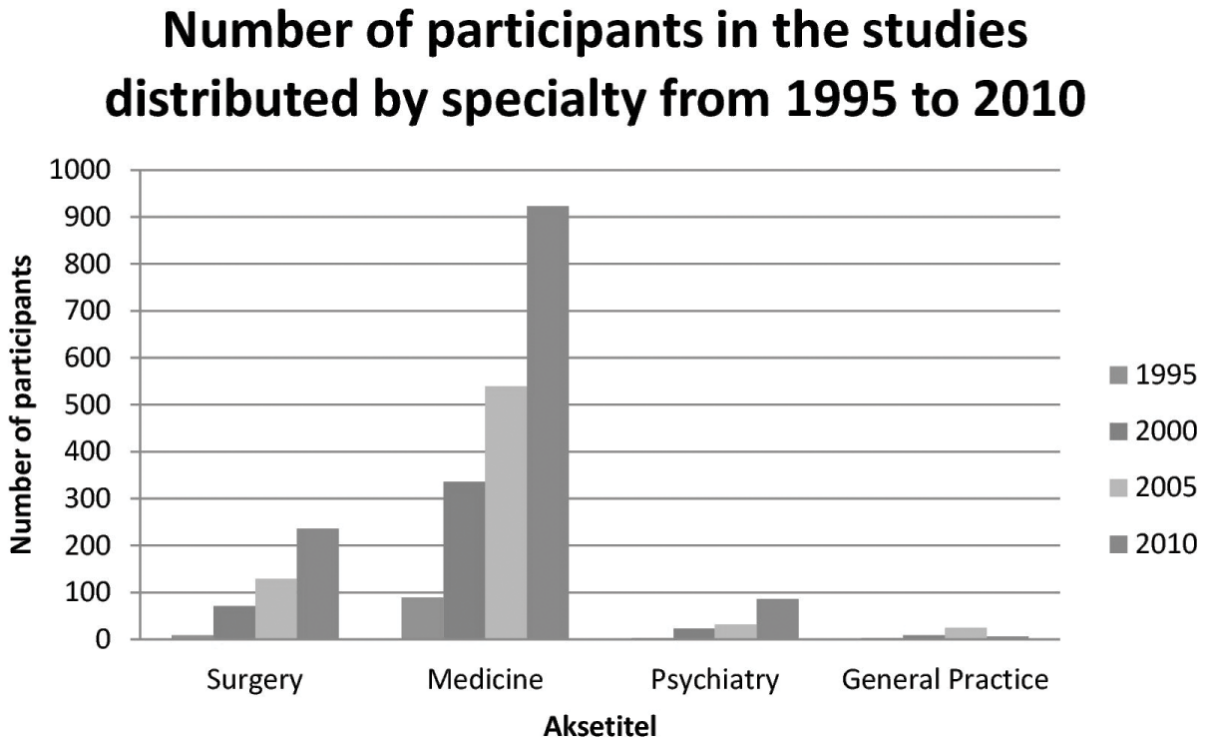
Number of participants in the studies distributed by specialty from 1995 to 2010.

When comparing the total number of patients in all specialties for the total study period, we found a variation between specialties, and not surprisingly we found a rise in total number of participants being included over the period (figure 5). We discovered that more patients were included in medical studies than surgical studies, an almost similar number of participants were included in surgical multicenter studies vs. psychiatric studies.

### Number of participating countries in multicentre studies

We found a rise in number of participating countries across the total study period both in total and in the specific specialty (in total 1995 n = 95, 2000 n = 418, 2005 n = 708, 2010 n = 1234). This variable was reported in 97.3% of the included studies.

### Type of intervention distributed by clinical specialty

We found an overweight of studies testing pharmaceuticals (in total, pharmaceuticals: n = 1,732, devices: n = 158, observational: n = 295, other: n = 369, with more medical studies reporting results on pharmaceuticals (Table 1). However, multicenter studies from surgical specialties accounted for more than 70% of the studies testing devices (Table 1).

**Table 1:** Distribution of intervention across specialties.

Intervention	Medicine	Surgery	Psychiatry	General Practice
**Device**	38(24%)	118 (74%)	2(1%)	0
**Pharmaceuticals**	1421(82%)	209(12%)	95(6%)	6(3%)
**Observational**	180(61%)	90(31%)	15(5%)	10(3%)
**Other**	270(73%)	37(10%)	34(9%)	28(8%)

Total number of studies, number in brackets showing percentages.

Additionally, we identified that studies testing pharmaceuticals accounted for 69% to 75% of studies being conducted in Europe, Asia, North and South America and Asia, and only 33% of the studies originating from Oceania.

## Discussion

In general, we found that the number of multicenter studies increased during the period from 1995 to 2010, with a larger share of multicenter studies been performed in Europe and North America compared with the rest of the world. Furthermore, the distribution between the different specialties showed that the medical specialties had the highest number of studies, but without substantial change in distribution between specialties over the years. When looking at the different interventions being tested in multicenter studies it was obvious that pharmacological interventions were overrepresented and primarily tested in medical studies followed by the testing of devices predominantly in surgical studies. The number of included patients as well as the number of participating departments increased during the time span, though the increase in studies was most evident in Europe and North America.

Multicenter studies may be a way of organizing clinical trials in order to increase power of the study, and to conduct studies of high scientific quality. This may establish the safety and efficacy of the tested intervention and produce results with clinical impact [10]. Multicenter studies may be applied within different clinical specialties testing different interventions. The organization of multicenter studies has been explored with focus on e.g. the issues of recruitment [11], and a specific focus on the establishment of efficient communication and collaboration [12].

Adequately sized randomized controlled trials are regarded as the gold standard in rigorous and robust clinical research. Participants may be recruited across a number of centers on a randomly assigned basis. One of the difficulties when carrying out clinical research is to have a high recruitment rate in order to have sufficient power. One way to overcome some of these difficulties could be to organize trials in multicenter studies.

We found that the number of multicenter studies increased during the period from 1995 to 2010. This interesting result hopefully reflects the rising awareness of the ethical issues within clinical decision making, as it should be based on best evidence from adequately sized randomized controlled trials [3]. When reflecting on this rise, we suggest that this may partly be because clinical researchers are becoming more attentive to the need for reporting the true value of the population's parameter, and not only relying on p-values. Certainly, the need for presenting confidence limits in order to demonstrate that the true population value is included may induce clinical researchers to design studies with larger samples; which may in fact be one of the reasons for establishing multi center studies.

When looking at the number of medical studies testing pharmaceuticals, there might be a need for not only testing a new drug and comparing it to placebo, but preferably to routine medication [13]. Obviously, this would result in a demand for inclusion of large numbers of participating patients because of a smaller difference in effect, which in turn may be another reason for joining multi center organization.

If pursuing the issue of inclusion, the increase in participating centers should be followed by an increase in included participants, hereby increasing sample size and power. However, we did not examine this relation further in this study, and therefore we cannot generalize that the increase in multi center studies did lead to studies reporting significant and clinically relevant results; both showing p-values and confidence intervals.

Some authors have discussed that one of the major concerns when planning and running multicenter studies was the increasing demands on cooperation and communication across departments, regions and countries [11]; a result which would deserve some attention, when planning large scale multicenter trials. It is obvious that some of the published trials were organized and run by professional clinical research organizations (CRO) in order to solve these problems, but we were not able to extract these data from the papers.

The medical specialties represented a larger number of studies organized as multicenter studies, as well as a large number of trials testing pharmaceuticals. Hence, it should be considered whether testing of pharmaceuticals may be easier organized in multicenter studies, or if the reason would be more frequent use of CROs to run the studies. A link between the structure and the intervention may be coincidental, but could be due to easier access to administrative and economic resources, or pharmaceuticals being easier to test than for instance surgical procedures [8]. Lack of resources is a major problem in randomized controlled trials where external funding may be crucial, thus a review found that 57% of published trials in dermatology were industry sponsored [14]. A company may be involved and supportive in different elements of a clinical trial: the design, the selection of researcher, the collection of data, the analysis of data and the reporting of findings, which may influence the trial and the reporting of it [15]. Therefore, these possible conflicts of interest must be declared and considered when interpreting the results. The differences between studies testing pharmaceuticals and devices were obvious, and we found that surgical multicenter studies accounted for more than 70% of the total studies testing devices. As with pharmaceutical studies obtaining funding, there is a similar possibility of sponsorship of device studies. A recent data study showed that the largest contributor to biomedical research in the US was medico-industry producing medical devices, followed by the National Institutes of Health [16]. Moreover, a study examining the clinicaltrials.gov database found that 81% of the reported industry-sponsored trials were reporting a drug intervention, 9% reported biologics/vaccines, and 8% reported studies testing devices [17]. This supports the fact that the high number of pharmaceutical studies in medical specialties may be sponsored by industry resources.

The difference between the continents was profound, showing that Europe and North America accounted for over 80% of the published multicenter studies, but probably with an increasing trend from Asia. The increase in Asia might be connected to an ease in establishing multicenter trials, and especially the rise in trials testing pharmaceuticals might be because of possible economic benefits related to for instance phase 3 trials.

Additionally, another perspective on the barriers related to research in for instance Africa might cover general aspects of a semi colonial approach to cooperation [18] and more specifically to challenges related to local culture, religion, and language [19]. Hence, the finding might not just be related to multicenter studies, but may reflect an overall problem related to clinical research in developing countries.

One of the limitations of this study was that we were not able to include a group for comparison, which means that the total number of trials being conducted the same period and in the same specialty is not scrutinized in our study. Therefore, our findings might reflect the development in clinical trials in general, and not just the development in multicenter organization. However, despite our data especially from some of the smaller clinical specialties were low in numbers, we were able to point at interesting differences in many of our variables. Doing a literature search as a mean of data retrieval was an obvious option for us, as there was no other alternative database where we could access the relevant data. Hence, we cannot be sure that our literature search fully identified all relevant trials for us to explore and examine the object of our study. However, we have retrieved data from published studies over a long period of time covering months that had similar publication rates as the rest of the year.

## Conclusions

We found an increase in numbers of clinical trials organized as multicenter studies primarily in Europe and North America. Furthermore, data revealed that medical specialties had a higher number of participating departments and number of included patients in multicenter studies compared with other specialties. However, we did not find large differences when comparing the increase in multicenter studies across the different clinical specialties. We also found that testing of pharmaceuticals, followed by testing of devices were the most widely used interventions in multicenter studies, and that pharmaceuticals were most often tested by medical specialties. This raises the question whether the larger proportion of multicenter studies within pharmaceuticals than devices may be related to funding from industry, which could be pursued in other studies.
